# Sea Buckthorn Fermented Milk with *Lactiplantibacillus plantarum* YHG-87 Mitigates Symptoms of DSS-Induced Ulcerative Colitis Disease in Mice

**DOI:** 10.3390/foods14213791

**Published:** 2025-11-05

**Authors:** Ning Ju, Xiaoliang Gu, Yuhong Ding, Yafeng Li, Shuai Guo

**Affiliations:** College of Food Science and Engineering, Ningxia University, Yinchuan 750021, China

**Keywords:** ulcerative colitis, sea buckthorn, *Lactiplantibacillus plantarum* YHG-87, fermented milk, colonic tissues

## Abstract

Ulcerative colitis (UC) is associated with gut microbiota imbalance, and probiotics may restore gut flora and improve intestinal health. Sea buckthorn, which is rich in antioxidants and anti-inflammatory compounds, may enhance these effects when combined with probiotics. In this study, we incorporated our screened strain *Lactiplantibacillus plantarum* YHG-87 (*L. plantarum* YHG-87) into sea buckthorn fermented milk to examine its additional benefits in a dextran sulfate sodium (DSS)-induced UC mouse model. Five groups (n = 6) were included: healthy control, DSS-induced colitis, regular fermented milk, sea buckthorn fermented milk, and sea buckthorn probiotic fermented milk (SC group). Results indicated that SC group significantly alleviated UC symptoms, such as weight loss, bloody stools, and colon shortening (*p* < 0.05), and these improvements were more pronounced than those observed in the sea buckthorn fermented milk group. Moreover, the SC group exhibited stronger anti-inflammatory and antioxidant effects, including reduced IL-6, increased IL-4 and IL-10, elevated glutathione, and reduced myeloperoxidase and malondialdehyde (*p* < 0.05). Additionally, the SC intervention led to notable shifts in gut microbial community composition. In conclusion, the addition of *L. plantarum* YHG-87 to sea buckthorn fermented milk provided enhanced protective effects against UC, suggesting that the combination of bioactive plant components with selected probiotics offers promising potential for UC prevention and therapy.

## 1. Introduction

Ulcerative colitis (UC) is a major type of inflammatory bowel disease (IBD), commonly characterized by diarrhea, bloody stools, and colon shortening [1]. Studies indicate that the development of UC is closely linked to abnormal intestinal epithelial cell function and a compromised intestinal mucosal barrier [2]. Without proper treatment, UC tends to recur, and ongoing intestinal damage raises the risk of colorectal cancer [3]. Current UC treatments mainly involve anti-inflammatory drugs and immunosuppressants, such as aminosalicylates, sulfasalazine, and corticosteroids [4]. While these drugs can relieve symptoms, they rarely cure the disease completely and may cause side effects or drug resistance with prolonged use. Therefore, the focus of current research is on exploring safe, effective, and long-term preventive and adjuvant therapies.

Recently, the role of probiotics in promoting health has gained significant attention, with research spanning IBD, irritable bowel syndrome, obesity, and allergies [5]. Probiotic fermented milk has been shown to benefit intestinal inflammation by improving digestion, lowering cholesterol [6], and modulating immune cells like T cells, dendritic cells, and macrophages [7]. *Lactiplantibacillus plantarum* (*L. plantarum*), in particular, has shown promise in alleviating UC by modulating immune responses, reducing oxidative stress, enhancing tight junction protein secretion, improving gut microbiota, and increasing short-chain fatty acid levels [7,8].

Sea buckthorn (*Hippophae rhamnoides L.*) is a medicinal and edible plant widely distributed in Asia and Europe, rich in vitamins (0.48–2.87%FW), polysaccharides (0.04–0.39%FW), organic acids (8.56 (g/kg), and polyphenols (28.97 mg GAE/g dw) [9], Its bioactive components, including polysaccharides, seed oil, and proanthocyanidins, have demonstrated anti-inflammatory, antioxidant, and immunomodulatory effects, and have been reported to alleviate UC symptoms [10]. In addition, sea buckthorn contains polysaccharides and polyphenols that may act as prebiotic substrates, selectively stimulating the growth and activity of beneficial bacteria, thus exerting a prebiotic-like effect. This suggests that sea buckthorn not only provides bioactive compounds but also may enhance the growth and function of probiotics, supporting gut health and UC prevention. Therefore, the abundant nutrients and health-promoting functions of sea buckthorn provide a strong rationale for its selection in this study. However, its high organic acid content contributes to a sour and astringent taste, limiting consumer acceptance. To overcome this limitation, we combined sea buckthorn with probiotic fermented milk, hypothesizing that this combination could not only improve palatability but also enhance synergistic protective effects against UC.

Previous studies have shown that sea buckthorn fermented milk, made by fermenting sea buckthorn puree and milk with *L. plantarum* YHG-87, contains significantly higher total phenol content than regular fermented milk. This not only improves flavor but also enhances in vitro antioxidant activity and lactic acid bacteria(LAB) count [11]. Relevant studies indicate that LAB can convert bound phenols in plants into free phenols, enhancing in vitro antioxidant activity [12]. Additionally, LAB produce substances such as short-chain fatty acids and amino acids through metabolism, which promote the expression of tight junction proteins in intestinal epithelial cells, thereby improving intestinal barrier function [13]. Therefore, combining bioactive substances from sea buckthorn with *L. plantarum* YHG-87 in fermented milk may positively affect UC prevention.

This research aims to assess the potential protective effects of sea buckthorn fermented milk with *L. plantarum* YHG-87 in a dextran sulfate sodium (DSS)-induced mouse model of UC. Key parameters, including disease activity index (DAI), colon length, body weight changes, cellular inflammatory factors, oxidative stress markers, and gut microbiota diversity and abundance, will be evaluated to further explore the fermented milk’s therapeutic effects and underlying mechanisms. This study provides evidence supporting the use of functional foods based on natural products and probiotics for UC prevention and adjunctive treatment, offering new insights for the development of safe and effective interventions.

## 2. Materials and Methods

### 2.1. Animals

Male specific pathogen-free (SPF) KM (Kunming mice) mice (age 8 weeks, weight 20 ± 2 g) were purchased from Chengdu dossy experimental animals Co., Ltd. (Chengdu, Sichuan, China). All mice were housed in a standard SPF environment in the animal house of the Sichuan Lilaisino Biotechnology Co., Ltd. (Chengdu, Sichuan, China). (granted the laboratory animal use permit, SYXK 2021-246). Six mice were maintained in each individually ventilated cage (temperature, 23 ± 3 °C; relatively humidity, 50 ± 10%; standard 12 h/12 h light/dark cycle). All mice were acclimatized one week before the formal experiment with free access to food and water ad libitum, and basic dietary feed by Chengdu dossy Laboratory Animal Co. (Chengdu, Sichuan, China). (Production License No. SCXK(Chuan)2019-028). All animal experimental protocols were strictly performed in accordance with the provisions of the National Institutes of Health of the United States, approved by the Ethics Committee of Sichuan Lilaisinuo Biotechnology Co. Ltd. (Chengdu, Sichuan, China) (LLSN-2023017).

One mouse in the DSS group died during modeling and was considered an adverse event, leading to missing end-point data. Therefore, the DSS group included five mice, while the other groups included six mice. All surviving animals were included in the analysis (Figure S1).

### 2.2. Preparation of Sea Buckthorn Fermented Milk with L. plantarum YHG-87, Sea Buckthorn Fermented Milk and Regular Fermented Milk

In this study, *L. plantarum* YHG-87, isolated from the traditionally fermented food “Jiangshui” in the Ningxia Hui Autonomous Region, was combined with a commercial fermented milk starter in a 2:1 ratio (3.48 × 10^9^ CFU/mL: 1.74 × 10^9^ CFU/mL, respectively). Pasteurized milk, sea buckthorn pulp (hereinafter referred to as “sea buckthorn,” *Hippophae rhamnoides* L., Ningxia Huaxinda Health Technology Co., LT, Yinchuan, Ningxia), and white sugar were mixed in approximate proportions of 87%, 6%, and 7% (*w*/*w*, Unit: g), respectively, and pasteurized at 70 °C for 30 min. After cooling, the mixture was reserved for later use. The activated bacterial *L. plantarum* YHG-87 culture was inoculated into the cooled mixture, and fermentation was carried out at 42 °C for 6 h to achieve a target pH of 4.5. Following fermentation, the product was refrigerated at 4 °C for overnight ripening, and fermented milk was prepared weekly during gavage. The regular and sea buckthorn fermented milk preparations followed the same procedure as that of the sea buckthorn fermented milk with *L. plantarum* YHG-87.

### 2.3. Trial Design

**Figure 1:**
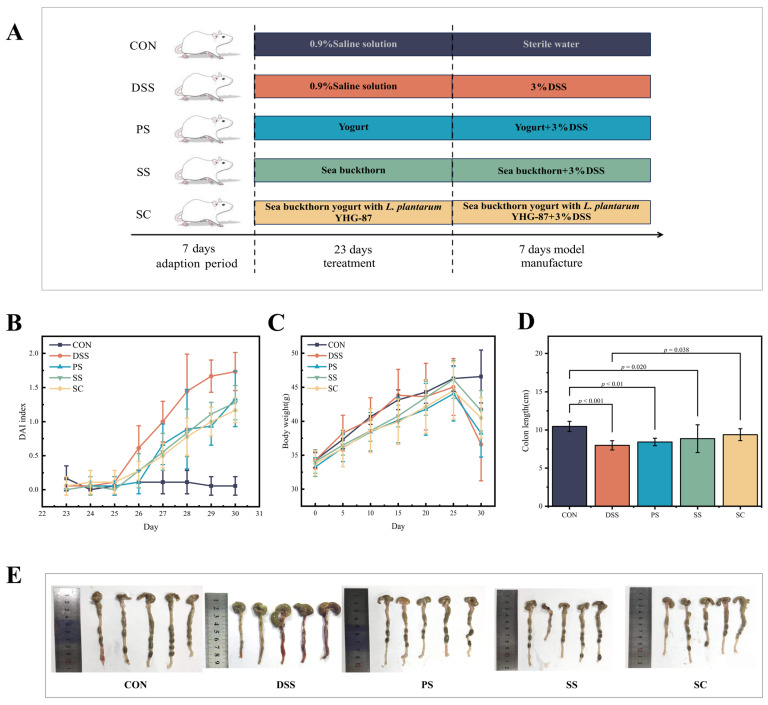
Sea buckthorn probiotic fermented milk administration attenuated the symptoms of DSS-induced colitis. (**A**) Schematic illustration of the experimental design. The “CON”, “DSS”, “PS”, “SS” and “SC” groups represented the healthy control, model, regular fermented milk, sea buckthorn fermented milk, and sea buckthorn fermented milk with *L. plantarum* YHG-87, respectively. (**B**) Disease activity index (DAI) scores at the end of the animal trial. (**C**) Body weight change in mice in the animal trial. (**D**) Colon length at the end of the animal trial. (**E**) Representative pictures of colon morphology of mice from the five treatment groups. Error bars represent standard deviation of the mean. Significant differences are represented by asterisks.

After a 7-day acclimatization period, the mice were randomly assigned to five treatment groups, each containing six mice: the healthy control group (CON), the model group (DSS), the regular fermented milk group (PS), the sea buckthorn fermented milk group (SS), and the sea buckthorn fermented milk with *L. plantarum* YHG-87 group (SC). The experiment lasted for 30 days. The CON and DSS groups were gavaged with 9% (*w*/*v*) saline, and the SC group were given a daily dose of 10 μL/g body weight, respectively. The modeling process began after 23 days of continuous gavage. A 3% (*w*/*v*) DSS solution was prepared in sterile water for modeling. The CON group continued to drink sterile water, while the other four groups received DSS solution. Simultaneously, the PS, SS, and SC groups continued their gavage treatments with the respective samples. The experiment concluded after 7 days of continuous gavage in all experimental groups (Figure 1A).

### 2.4. Disease Activity Index (DAI)

Mice were weighed daily, and their general condition was monitored. Fecal characterization and occult blood were scored, along with disease activity scoring. The DAI was determined daily based on body weight loss, stool consistency, and fecal occult blood (Determination by o-toluidine method). The DAI was calculated as follows: (Weight loss score + Stool consistency score + fecal occult blood score)/3, with higher values indicating more severe intestinal inflammation in mice (Table 1).

### 2.5. Colon Length Measurement

A section of the mouse colon was treated with cold physiological saline. Excess moisture was then removed with clean filter paper. The colon tissue was then spread flat, and its length was measured using a ruler.

### 2.6. Histopathological Analysis

A 0.5 cm colon tissue sample was processed using an automatic dehydrator, followed by embedding and sectioning. The sections were subsequently stained with hematoxylin and eosin (H&E) and imaged with a digital slide scanner. The histological score was determined according to criteria in Table 2.

### 2.7. Changes in Inflammatory Cytokines in the Serum

After collecting blood from the mice, the samples were centrifuged at 3500 rpm for 10 min at 4 °C, and the supernatant was isolated. Inflammatory cytokine levels, including Interleukin-4 (IL-4), Interleukin-10 (IL-10), Interleukin-6 (IL-6), and Tumor Necrosis Factor-α (TNF-α), were quantified using an ELISA kit (Shanghai Zhuocai Biotechnology Co., Ltd., Shanghai, China). An automatic plate washer(PW-480, Shenzhen Huisong Technology Development Co., Ltd., Shenzhen, Guangdong, China) was used for washing, and readings were taken using a SpectraMAX Plus384 (Meigu Molecular Instruments Co., Ltd., Shanghai, China).

### 2.8. Tissue Oxidative Damage Assessment

After obtaining the serum from the mice, malondialdehyde (MDA) levels were measured using an MDA assay kit (Nanjing Jianjian Bioengineering Research Institute, Nanjing, Jiangsu, China). Glutathione (GSH) and myeloperoxidase (MPO) levels were measured using the same methods as those used for inflammatory cytokines (Shanghai Zhuocai Biotechnology Co., Ltd., Shanghai, China).

### 2.9. Fecal DNA Extraction, Sequencing and Analysis

150 mg of mouse feces was transferred into a sterile centrifuge tube. Then, 1 mL of lysis buffer (PBS) was added, and the mixture was vortexed. 20 µL of proteinase K (final concentration ~20 mg/mL) was added, followed by another vortexing. The sample was incubated at 55 °C in a water bath for 30–60 min, vortexing every 10 min to ensure thorough mixing. Afterward, an equal volume of phenol-chloroform-isoamyl alcohol (25:24:1) was added, and the sample was mixed thoroughly before centrifugation at 12,000× *g* for 10 min. Twice the volume of cold anhydrous ethanol and one-tenth the volume of sodium acetate (3M, pH 5.2) were added. The sample was mixed thoroughly and incubated at −20 °C for 30 min. The DNA precipitate was collected by centrifugation at 12,000× *g* for 10 min. The precipitate was washed with 70% ethanol, dried, and dissolved in TE buffer or sterile water. Low-speed centrifugation (500–1000× *g* for 1–2 min) was performed to remove large particles, leaving the supernatant. DNA integrity and size were assessed using 1.2% agarose gel electrophoresis.

The 16S rRNA gene was amplified using primers 341F (5′-CCTACGGGNGGCWGCAG-3′) and 806R (5′-GGACTACHVGGGTATCTAAT-3′), targeting the V3-V4 region. The DNA libraries were prepared using the TruSeq Nano DNA LT Library Prep Kit (Illumina, San Diego, CA, USA), and sequencing was performed on an Illumina platform, with paired-end 2 × 300 bp reads. Negative controls (NTC) were included by substituting sterile water for the sample DNA. After sequencing, the raw data were initially screened based on sequence quality. Problematic samples were re-sequenced or supplemented as necessary to ensure data integrity. Following quality control, the raw sequences were processed based on their index and barcode information, and barcode sequences were removed. Sequence denoising and clustering were performed using the QIIME2 dada2 pipeline [14,15], which generates Amplicon Sequence Variants (ASVs), instead of traditional Operational Taxonomic Units (OTUs), for a more accurate microbial community profile. The use of ASVs provides a finer-resolution classification of microbial sequences, enhancing the accuracy of diversity analysis. The species composition of each sample was analyzed at different taxonomic levels, providing a comprehensive overview of the gut microbiome. Alpha diversity indices, such as Chao1, Shannon, and Simpson, were calculated based on the distribution of ASVs across samples. Rarefaction curves were generated to confirm sufficient sequencing depth, ensuring that the results were not influenced by sequencing bias. To assess beta diversity, a distance matrix was calculated at the ASV level, and various unsupervised methods, such as ordination (PCoA) and clustering, were applied to evaluate differences in microbial communities across the samples. Appropriate statistical tests, including PERMANOVA, were performed to determine the significance of these differences. At the species classification level, both unsupervised and supervised methods were used to analyze the abundance composition of species across different samples. We then constructed a network based on the distribution of species in each sample, calculating topological indices to identify key species that may play critical roles in the microbiome. All experiments were conducted in triplicate to ensure the robustness and reliability of the data. The results were further validated using multiple statistical analyses, including Kruskal–Wallis tests for alpha diversity, PERMANOVA for beta diversity, and differential abundance analyses (LEfSe and Wilcoxon rank-sum tests), to ensure the consistency and robustness of the findings.

### 2.10. Statistical Analyses

Statistical analysis was conducted using IBM SPSS Statistics 22.0. For repeated measures, appropriate methods such as repeated-measures analysis of variance (RM-ANOVA) and linear mixed-effects models (LMMs) were used to account for within-subject correlation and unequal sample sizes. For single-time-point analyses, one-way ANOVA was applied. Post hoc multiple comparisons were performed using the LSD test when variance homogeneity was assumed, and Tamhane’s T2 test when it was not. Data were expressed as mean  ±  standard deviation. *p* <  0.05 was considered statistically significant.

## 3. Results

### 3.1. Effects of Sea Buckthorn Fermented Milk with L. plantarum YHG-87 on DAI Score and Colon Length in Mice

The DAI scores were plotted based on changes in rectal bleeding, stool consistency, and weight loss in mice (Figure 1B, Tables S1–S5). At the end of the intervention, DAI scores in the DSS group were significantly higher than those in the CON group (*p* < 0.001). Meanwhile, in the DAI score, the PS group, SS group and the SC group showed significant differences from the DSS group (*p* < 0.05). Thus, fermented milk administration helps alleviate the underlying symptoms reflected in the DAI score. Regarding body weight, the DSS group showed a significant decrease in body weight change compared to the CON group (*p* < 0.01) (Figure 1C, Table S6). The body weight changes in the PS, SS, and SC groups were significantly higher than those in the DSS group (*p* < 0.05). However, no significant difference was observed among the fermented milk groups (*p* > 0.05). In this experiment, the average colon length in the DSS group was significantly shorter than in the CON group (*p* < 0.01) (Figure 1D,E). The colon length in the SC groups significantly increased compared to the DSS group.

### 3.2. Histopathological Analysis of Colon Tissue

**Figure 2:**
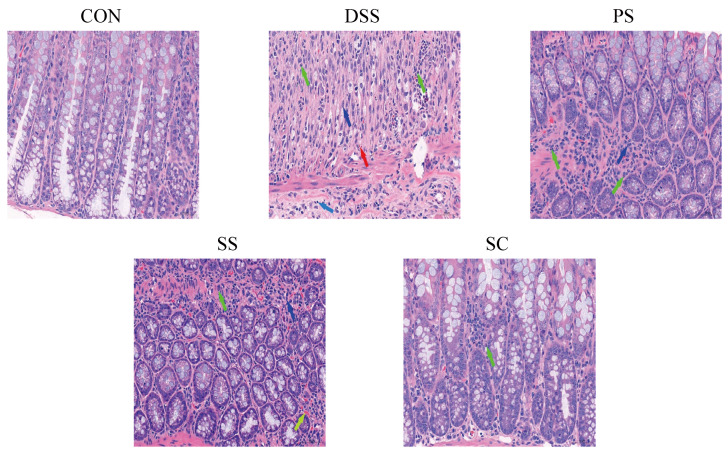
Sea buckthorn probiotic fermented milk administration protected against DSS-induced colon tissue damage (Hematoxylin and eosin staining microscopic images). Green, light blue, blue, red, and yellow arrows denote. neutrophils, lymphocytes, fibroblasts, new capillaries, and vascular congestion, respectively. (CON: the healthy control group, DSS: the model group, PS: the regularly fermented milk group, SS: the sea buckthorn fermented milk group, SC: the sea buckthorn with *L. plantarum* YHG-87 fermented milk group).

DSS-induced UC is characterized by intestinal mucosal damage, colon epithelial cell injury, and inflammatory cell infiltration, representing typical morphological and pathological changes [16,17]. Therefore, we used colon tissue pathology as an evaluation criterion in this experiment. H&E staining (Figure 2) showed that the CON group’s colon tissue structure was intact with normal epithelial cell morphology, no significant degeneration, necrosis, or detachment, and a normal number of goblet cells. In contrast, the DSS group’s mucosal layer exhibited varying degrees of degeneration and necrosis, with tissue hyperplasia and some inflammatory cell infiltration in necrotic areas. Some SS and PS group samples showed minimal inflammatory cell infiltration and mild fibrous tissue hyperplasia. Following sea buckthorn probiotic fermented milk intervention, the mucosal, submucosal, muscular, and serosal layers of the colon tissues remained intact, with no significant degeneration, necrosis, or detachment, indicating a protective effect from prior consumption.

### 3.3. Changes in Serum Cytokines Levels

**Figure 3:**
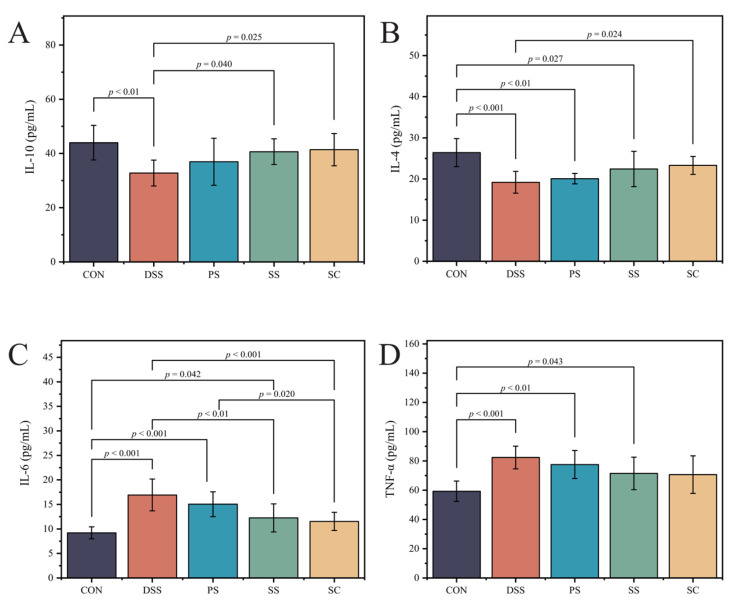
The fermented milk intake alleviated DSS-induced changes in serumPro-/anti-inflammatory cytokine levels (**A**) Interleukin-10 (IL-10), (**B**) Interleukin-4 (IL-4), (**C**) Interleukin-6 (IL-6), (**D**) Tumor necrosis factor-α (TNF-α). Error bars represent standard deviation of the mean. Significant differences are represented by asterisks. (CON: the healthy control group, DSS: the model group, PS: the regular fermented milk group, SS: the sea buckthorn fermented milk group, SC: the sea buckthorn fermented milk with *L. plantarum* YHG-87 group).

This study investigated the effects of sea buckthorn fermented milk with *L. plantarum* YHG-87 on inflammatory markers in DSS-induced UC. We measured pro-inflammatory factors (IL-6 and TNF-α) and anti-inflammatory factors (IL-4 and IL-10) to evaluate the immune response. Compared with the CON group, IL-4 (Figure 3A, Table S7) and IL-10 (Figure 3B, Table S8) levels were significantly reduced in the DSS group (*p* < 0.05), while IL-6 (Figure 3C, Table S9) and TNF-α (Figure 3D, Table S10) levels were significantly increased (*p* < 0.05). After intervention, IL-4 and IL-10 levels were significantly higher and IL-6 levels significantly lower in the SC group compared with the DSS group (*p* < 0.05). In contrast, TNF-αshowed only a downward trend in the SC group but did not reach statistical significance (*p* > 0.05). No significant changes in IL-10 were observed among the fermented milk groups compared with the CON group, suggesting that fermented milk intervention may help maintain baseline IL-10 levels. These results indicate that sea buckthorn fermented milk with *L. plantarum* YHG-87 exerts a stronger modulatory effect on cytokines, particularly by enhancing IL-4 and IL-10 and suppressing IL-6.

### 3.4. Changes in Oxidative Stress Levels

**Figure 4:**
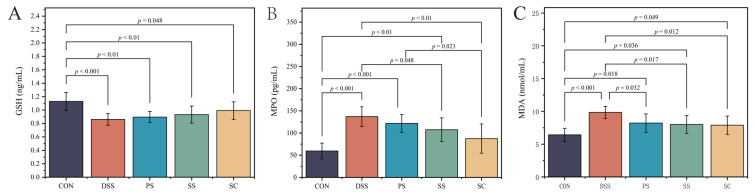
Effect of sea buckthorn probiotic fermented milk supplementation on oxidative stress marker. (**A**) Glutathione (GSH), (**B**) Myeloperoxidase (MPO) and (**C**) Malondialdehyde (MDA). Error bars represent standard deviation of the mean. Significant differences are represented by asterisks. (CON: the healthy control group, DSS: the model group, PS: the regular fermented milk group, SS: the sea buckthorn fermented milk group, SC: the sea buckthorn fermented milk with *L. plantarum* YHG-87 group).

Glutathione (GSH), myeloperoxidase (MPO), and malondialdehyde (MDA) were used as indicators of tissue oxidative damage (Figure 4). The results showed that compared to CON group, the DSS group had significantly lower GSH(Figure 4A, Table S11) levels and significantly higher MPO(Figure 4B, Table S12) and MDA(Figure 4C, Table S13) levels (*p* < 0.05). Additionally, MDA levels were significantly reduced in all three fermented milk intervention groups compared to the DSS group (*p* < 0.05). Although GSH levels increased in the SC group compared to the DSS group, the difference was not statistically significant (*p* > 0.05). The SC group had significantly lower MPO levels compared to the DSS group (*p* < 0.05). No significant differences were observed between the fermented milk groups for the other two indicators, except for MPO. However, the SC group displayed some advantages (*p* > 0.05). These results indicate that sea buckthorn fermented milk with *L. plantarum* YHG-87 has the strongest antioxidative and anti-inflammatory effects.

### 3.5. Gut Microbiota Composition

**Figure 5:**
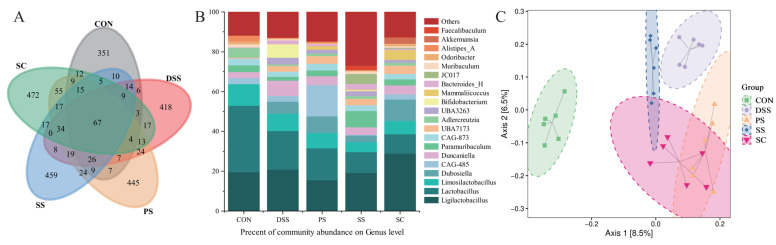
Effect of sea buckthorn probiotic fermented milk on the gut Microbiota composition. (**A**) The Venn network plot illustrates the common and unique microorganisms identified in comparisons among different groups. (**B**) Genus-level gut microbiota profile. (**C**) Principal coordinate analysis (PCoA; Bray–Curtis distance) score plot of mouse gut microbiota. (CON: the healthy control group, DSS: the model group, PS: the regular fermented milk group, SS: the sea buckthorn fermented milk group, SC: the sea buckthorn fermented milk with *L. plantarum* YHG-87 group).

To visually represent OTU similarity and overlap among samples, Venn diagrams were used for statistical analysis of each experimental group’s OTUs. The results showed that the five experimental groups shared 67 OTUs. The SC group had 472 unique OTUs, followed by 459 in the SS group, 445 in the PS group, 351 in the CON group, and 418 in the DSS group (Figure 5A). Further taxonomic analysis at the genus level revealed compositional variations among groups (Figure 5B). Genera such as *Bacteroides*, *Adlercreutzia*, *Akkermansia*, and *Ligilactobacillus* showed different relative abundances across treatments. Compared with the DSS group, fermented milk interventions—particularly the SC group—resulted in clear shifts in microbial community structure. PCoA demonstrated distinct separation between the DSS and SC groups, while the three fermented milk groups showed partial overlap, suggesting similar yet differentiated community patterns (Figure 5C, Table S14). Overall, these results indicate that supplementation with *L. plantarum* YHG-87 in sea buckthorn fermented milk altered the gut microbial composition and may contribute to maintaining intestinal homeostasis.

## 4. Discussion

Intestinal microecological imbalance is considered a key pathogenic factor in the pathophysiology of UC [18,19]. In UC patients, beneficial bacteria decrease while pathogenic bacteria increase, leading to impaired intestinal barrier function and excessive immune responses. Probiotics, as biotherapeutics that balance gut microbiota and improve the intestinal environment, have gained significant attention in recent years [20]. Sea buckthorn is a medicinal and edible plant rich in vitamins, flavonoids, and active compounds [9], possessing antioxidant, anti-inflammatory, and immunomodulatory properties [21]. Studies show that sea buckthorn polysaccharides and proanthocyanidins benefit gut health by inhibiting inflammatory factor expression and alleviating intestinal inflammation [10]. Thus, combining sea buckthorn with *L. plantarum* YHG-87 may synergistically modulate gut microbiota, enhance mucosal barrier function, and alleviate inflammatory responses, providing a protective effect against UC. This study combined sea buckthorn with *L. plantarum* YHG-87 to evaluate the therapeutic effects of sea buckthorn fermented milk with *L. plantarum* YHG-87 in a DSS-induced UC model.

In preliminary experiments, we investigated the mixed fermentation process of sea buckthorn fermented milk with *L. plantarum* YHG-87. Results showed that adding *L. plantarum* YHG-87significantly increased key flavor compounds in fermented milk, such as cyclohexanone, 2-heptanol, 2,3-butanedione, and trans-2-hexenal. Incorporating sea buckthorn introduced 11 volatile compounds, including acids, esters, ketones, alcohols, α-pinene, 2,5-dimethylfuran, and 2,3,5-trimethylpyrazine, enhancing the fermented milk’s flavor profile. Furthermore, adding sea buckthorn not only enhanced flavor but also significantly increased *L. plantarum* content. The experimental results showed that sea buckthorn fermented milk with *L. plantarum* YHG-87 not only significantly enhanced *L. plantarum* YHG-87 growth but also exhibited the highest levels of total phenolic content and in *vitro* antioxidant activities, including DPPH radical scavenging, ABTS radical scavenging, and ferric ion reducing power, among the tested products [11].

Based on previous research, this study further explored the effects of sea buckthorn fermented milk with *L. plantarum* YHG-87 on DSS-induced UC. DSS disrupts the intestinal barrier, making the gut susceptible to harmful microorganisms, which leads to symptoms like weight loss, shortened colon length, and bloody stool [22]. Results indicated that, compared to the DSS group, SC group mice treated with sea buckthorn fermented milk with *L. plantarum* YHG-87 showed significant improvements in weight, bleeding scores, and colon length, particularly in alleviating weight loss, demonstrating its preventive and alleviating effects. Similar studies have shown that probiotic preparations protect against UC, probiotics had been shown to alleviate symptoms of UC, including weight loss, diarrhea, blood in the stool, and a shortened colon length, while also restoring intestinal microecological homeostasis, improving gut barrier function, modulating the intestinal immune response, and attenuating intestinal inflammation [23]. For example, Georgios et al. reported that *Lactobacillus rhamnosus* reduce UC symptoms by modulating gut microbiota and enhancing intestinal barrier function [24]. Regarding colon length, the PS, SS, and SC groups all showed some beneficial effects compared with the DSS group, but only the SC group showed a significant increase in colon length, indicating that sea buckthorn fermented milk with *L. plantarum* YHG-87 positively affected colon shortening. Colon shortening was a hallmark of UC. The experiments demonstrated that several treatment groups had positive effects on body weight relative to the model group. In terms of colon length, the SC group showed a more significant effect, although other groups showed positive effects, albeit without significant changes. Similarly, DAI scores yielded comparable results. Mantel et al. [25] fermented whole milk with the probiotic *Propionibacterium freudenreichii*, demonstrating its significant reduction in UC clinical signs in a mouse model of colitis. This study further confirms that adding sea buckthorn components can enhance this protective effect. This effect may be attributed to the polyphenols and antioxidants present in sea buckthorn, which enhance the integrity of the intestinal mucosal barrier and immune function, thereby combating intestinal inflammation.

During intestinal inflammation, the balance between pro-inflammatory and anti-inflammatory factors is crucial for disease progression and recovery. Increased levels of pro-inflammatory factors like IL-6 and TNF-α [26] and decreased levels of anti-inflammatory factors like IL-4 and IL-10 are key markers of disease progression. IL-4 regulates the expression of pro-inflammatory factors in immune responses and alleviates UC by increasing intestinal mucus thickness through goblet cell modulation [27]. IL-10 exerts anti-inflammatory effects by inhibiting the secretion of pro-inflammatory factors. This study found that after sea buckthorn fermented milk with *L. plantarum* YHG-87 intervention, levels of anti-inflammatory factors IL-10 and IL-4 in mice significantly increased, while the level of pro-inflammatory factor IL-6 significantly decreased. Although TNF-α did not show a significant change, it exhibited a downward trend. These findings suggest that sea buckthorn fermented milk with *L. plantarum* YHG-87 may regulate the balance of inflammatory factors. Previous studies have shown that probiotics can balance immune responses by modulating inflammatory factor expression, thereby alleviating intestinal inflammation [28]. Moreover, studies using anti-TNF-α and anti-IL-6 monoclonal antibodies have demonstrated that reducing TNF-α and IL-6 levels significantly alleviates colonic damage in colitis [26]. These findings further support the potential of sea buckthorn fermented milk with *L. plantarum* YHG-87 for preventing and treating UC. Additional studies support this finding. For example, Ladda et al. [29] demonstrated that probiotics can reduce IL-6 and TNF-α expression by modulating the NF-κB signaling pathway, thereby alleviating intestinal inflammation. Consistent with these findings, sea buckthorn fermented milk with *L. plantarum* YHG-87 seems to better maintain immune balance in the gut and reduce abnormal elevations in inflammatory factors. Additionally, UC is often characterized by intestinal mucosal damage, epithelial cell injury, and inflammatory cell infiltration. In this study, H&E staining revealed significant structural damage in the colonic tissues of DSS-treated mice. In contrast, mice treated with sea buckthorn fermented milk with *L. plantarum* YHG-87 showed intact intestinal structures and significantly reduced inflammatory cell infiltration. This suggests that sea buckthorn fermented milk with *L. plantarum* YHG-87 may repair intestinal mucosa and enhance gut barrier function, likely due to the anti-inflammatory properties of *L. plantarum* YHG-87. Previous studies have shown that certain probiotics, such as *Lactobacillus* and *Bifidobacterium*, maintain gut barrier function by increasing tight junction protein expression, thereby reducing inflammatory responses [30]. *L. plantarum* YHG-87 combined with the antioxidant components in fermented milk and sea buckthorn showed a strong synergistic effect in this respect.

Oxidative stress is a crucial mechanism in the occurrence and development of UC [31]. In this study, antioxidant GSH levels in DSS group mice significantly decreased, while MPO and MDA levels significantly increased, indicating severe tissue damage and oxidative stress response. After intervention with sea buckthorn fermented milk containing *L. plantarum* YHG-87, GSH levels in the SC group showed a slight increase compared to the DSS group, but the difference was not statistically significant, while MPO and MDA levels decreased significantly. Although the SC group showed a smaller advantage over the PS and SS groups at the MDA level, it demonstrated an advantage at both the MPO levels, with significantly higher MPO levels than the SS group, indicating a positive antioxidant effect. Previous studies have shown that probiotics effectively reduce oxidative stress by promoting antioxidant enzyme expression and reducing free radical production, thereby alleviating tissue damage. Similarly, Nithya et al. [32] discussed probiotic fermented foods, such as Axone and Chathur, which are fermented with *Lactobacillus* and had been shown to inhibit oxidative stress and modulated the immune response to reduce intestinal damage. This study further suggests that incorporating sea buckthorn components may enhance the antioxidant effects of *L. plantarum* YHG-87. Previous studies, including Ge et al., had demonstrated that the addition of sea buckthorn to fermented milk might enhance the activity of LAB by providing bioactive compounds such as polysaccharides and polyphenols, which served as fermentable substrates for LAB [33]. In our study, *L. plantarum* YHG-87, a LAB that metabolizes carbohydrated to produce lactic acid, was combined with sea buckthorn to ferment fermented milk. We hypothesized that the polysaccharides and polyphenols in sea buckthorn might further promote the growth and metabolic activity of *L. plantarum* YHG-87, leading to enhanced lactic acid production and increased health benefits, including improvements in gut microbiota, oxidative stress, and inflammation, which were observed in our DSS-induced UC model.

IBD is closely related to intestinal microbiota composition [34], and changes in microbial diversity and abundance can affect colitis occurrence and progression [35,36]. In this study, distinct shifts in the gut microbial community structure were observed after the administration of sea buckthorn fermented milk with *L. plantarum* YHG-87. Compared with the DSS group, the SC group showed compositional differences at the genus level, involving taxa such as *Akkermansia*, *Ligilactobacillus*, *Adlercreutzia*, *Dubosiella*, and *Bacteroides*. Previous studies have shown that certain *Akkermansia* species modulate mucosal immunity and promote short-chain fatty acid production [37,38]. *Ligilactobacillus* contributes to intestinal barrier integrity and the regulation of inflammatory cytokines [39,40]. *Adlercreutzia* has been reported to metabolize polyphenols such as resveratrol and exert antioxidant effects [41,42]. *Dubosiella* has been suggested to alleviate oxidative stress and improve mucosal barrier function through the production of propionate and L-lysine [43,44]. In addition, *Bacteroides* species are commonly associated with polysaccharide degradation and maintenance of gut metabolic balance [43,44]. However, because 16S rRNA sequencing provides genus-level rather than species-level resolution, the functional implications of these community changes cannot be definitively determined. Therefore, the observed alterations should be interpreted cautiously, and further studies using higher-resolution techniques (e.g., metagenomic sequencing or strain-specific PCR) are warranted to verify whether probiotic supplementation truly enhances beneficial bacterial populations and to clarify the precise roles of these genera in gut health. In summary, supplementation with *L. plantarum* YHG-87 in sea buckthorn fermented milk effectively alleviated UC-related symptoms, enhanced antioxidant and anti-inflammatory responses, and modulated gut microbial composition. These results suggest that combining bioactive plant compounds with probiotics may provide synergistic benefits for maintaining intestinal homeostasis and preventing colitis. Nevertheless, although distinct microbial shifts were observed, further studies are needed to confirm whether these changes correspond to actual increases in beneficial bacteria and to elucidate the underlying mechanisms linking microbial modulation with host protection.

Although this study shows that sea buckthorn fermented milk with *L. plantarum* YHG-87 has a significant effect in alleviating DSS-induced UC, there are some limitations. First, the study primarily examined the effects of sea buckthorn fermented milk with *L. plantarum* YHG-87 in a mouse model; further research is needed to assess its efficacy in humans. Second, the study did not comprehensively analyze the mechanisms behind changes in gut microbiota, such as the links between specific microbiota and inflammatory responses. Therefore, future research should investigate the regulatory mechanisms of sea buckthorn fermented milk with *L. plantarum* YHG-87 on specific microbiota and explore its action pathways using multi-omics approaches. Additionally, the study did not explore the specific mechanisms of action and interactions of the various active components in sea buckthorn fermented milk with *L. plantarum* YHG-87. Future research should concentrate on elucidating the molecular mechanisms, especially the signaling pathways involving the combined effects of sea buckthorn components and *L. plantarum* YHG-87. Sea buckthorn fermented milk with *L*. *plantarum* YHG-87 showed potential in alleviating UC symptoms by reducing inflammation, improving oxidative stress, and promoting gut health. This combination offered a promising approach for UC treatment.

## 5. Conclusions

In conclusion, sea buckthorn fermented milk with *L. plantarum* YHG-87 demonstrates significant potential in the prevention and treatment of UC by alleviating clinical symptoms, modulating inflammatory and antioxidant markers, repairing colon tissue damage, and promoting beneficial gut microbiota. These effects suggest a synergistic benefit from combining the antioxidant and anti-inflammatory properties of sea buckthorn with *L. plantarum* YHG-87, offering a promising therapeutic strategy for UC management.

## Figures and Tables

**Table 1:** Criteria for scoring disease activity index.

Score	Weight Loss (%)	Stool Consistency	Occult Blood or Gross Bleeding
0	<1	Normal	Negative
1	1–5	Loose stool	Negative
2	5–10	Loose stool	Hemoccult positive
3	10–15	Diarrhea	Hemoccult positive
4	>15	Diarrhea	Gross bleeding

Note: disease activity index = (combined score of weight loss, stool consistency, and occult blood or gross bleeding)/3.

**Table 2:** Histological grading of colitis.

Score	Severity of Inflammation	Depth of Injury	Crypt Damage
0	None	None	None
1	Slight	Mucosa	Basal 1/3 damaged
2	Moderate	Mucosa and submucosa	Basal 2/3 damaged
3	Severe	Transmural	Only surface epithelium intact
4	–	–	Entire crypt and epithelium lost

Note: the histological score for each mouse was a sum of the score of severity of inflammation, depth of injury, and crypt damage.

## Data Availability

The data presented in this study are available on request from the corresponding author.

## Supplementary Materials

The following supporting information can be downloaded at: https://www.mdpi.com/article/10.3390/foods14213791/s1, Figure S1: Schematic diagram of a simple experimental design.; Table S1 DAI scores after modeling (CON); Table S2: Changes in body weight of mice in each group after modeling (±SD,DSS); Table S3: Changes in body weight of mice in each group after modeling (±SD,PS); Table S4: Changes in body weight of mice in each group after modeling (±SD,SS); Table S5: Changes in body weight of mice in each group after modeling (±SD,SC); Table S6: Length of colon (±SD); Table S7: Changes in IL-4 concentration in serum of mice in each group (±SD); Table S8: Changes in IL-10 concentration in serum of mice in each group (±SD); Table S9: Changes in serum concentrations of IL-6 in mice in each group (±SD); Table S10: Changes in TNF-α concentration in serum of mice in each group (±SD); Table S11: Changes in GSH concentration in serum of mice in each group (±SD); Table S12: Changes in MPO concentration in serum of mice in each group (±SD); Table S13: Changes in MDA content in serum of mice in each group (±SD); Table S14: Permutational multivariate analysis of variance.

## Abbreviations

The following abbreviations are used in this manuscript:

UC	Ulcerative colitis
*L. plantarum*	*Lactiplantibacillus plantarum*
DSS	dextran sulfate sodium
IBD	inflammatory bowel disease
DAI	disease activity index
SPF	specific pathogen-free
KM	Kunming mice
IL-4	Interleukin-4
IL-10	Interleukin-10
IL-6	Interleukin-6
TNF-α	Tumor Necrosis Factor-α
MDA	malondialdehyde
GSH	glutathione
MPO	myeloperoxidase
OTUs	operational taxonomic units
