# Supplementary material for: Sea Buckthorn Fermented Milk with Lactiplantibacillus plantarum YHG-87 Mitigates Symptoms of DSS-Induced Ulcerative Colitis Disease in Mice
Source: Foods. 2025 Nov 5;14(21):3791. doi: 10.3390/foods14213791 (PMC12610435; doi:10.3390/foods14213791)
Supplement: Supplementary file 1 [file foods-14-03791-s001.zip › Data table.pdf]

Table S1 DAI scores after modeling (CON)

Day	N	DAI Score	Day
23	6	0.16667±0.18257	23
24	6	0±0	24
25	6	0.05556±0.13608	25
26	6	0.11111±0.17213	26
27	6	0.11111±0.17213	27
28	6	0.11111±0.17213	28
29	6	0.05556±0.13608	29
30	6	0.05556±0.13608	30

Table S2 Changes in body weight of mice in each group after modeling (±SD,DSS)

Day	N	$\bar{x}$±SD	Day
0	6	34.25±1.27711	0
5	6	38.26667±2.6212	5
10	6	40.25±3.20297	10
15	6	43.83333±3.80771	15
20	6	43.63333±4.91067	20
25	6	45.05±4.17313	25
30	5	36.58±5.34341	30

Table S3 Changes in body weight of mice in each group after modeling (±SD,PS)

Day	N	$\bar{x}$±SD	Day
0	6	33.26667±1.38516	0
5	6	36.1±2.05329	5
10	6	38.45±2.82754	10
15	6	40.16667±3.34345	15
20	6	41.76667±3.80876	20
25	6	44.05±4.00537	25
30	5	38.24±3.50471	30

Table S4 Changes in body weight of mice in each group after modeling (±SD,SS)

Day	N	$\bar{x}$±SD	Day
0	6	34.08333±1.73369	0
5	6	36.5±1.48459	5
10	6	38.6±1.53493	10
15	6	40.78333±1.60427	15
20	6	43.51667±2.62862	20
25	6	46.15±2.77903	25
30	6	41.55±2.96429	30

Table S5 Changes in body weight of mice in each group after modeling (±SD,SC)

Day	N	$\bar{x}$±SD	Day
0	6	33.8±1.82099	0
5	6	36.03333±2.73691	5
10	6	38.58333±3.1915	10
15	6	39.91667±3.35405	15
20	6	42.15±3.91344	20
25	6	44.51667±4.20401	25
30	6	40.48333±3.00161	30

Table S6 Length of colon (±SD)

Group	N	±SD
CON	6	10.55±0.63797
DSS	5	7.98±0.60581
PS	5	8.42±0.49193
SS	6	8.98333±1.65338
SC	6	9.4±0.70427

Table S7 Changes in IL-4 concentration in serum of mice in each group (±SD)

Group	Sample size (cases)	IL-4 (pg/mL)
CON	6	26.41367±3.4064
DSS	5	19.20067±2.64627
PS	5	20.08517±1.278
SS	6	22.42733±1.278
SC	6	23.299±2.18356

Table S8 Changes in IL-10 concentration in serum of mice in each group (±SD)

Group	Sample size (cases)	IL-10 (pg/mL)
CON	6	43.991±6.40268
DSS	5	32.78467±4.7753
PS	5	36.9435±8.67663
SS	6	40.6475±4.73382
SC	6	41.41583±5.93852

Table S9 Changes in serum concentrations of IL-6 in mice in each group (±SD)

Group	Sample size (cases)	IL-6 (pg/mL)
CON	6	9.20783±1.22515
DSS	5	16.91783±3.24516
PS	5	15.04417±2.53375
SS	6	12.25±2.8657
SC	6	11.53467±1.85783

Table S10 Changes in TNF-α concentration in serum of mice in each group (±SD)

Group	Sample size (cases)	TNF-α (pg/mL)
CON	6	59.254±6.99703
DSS	5	82.38033±7.74543
PS	5	77.54067±9.59068
SS	6	71.46083±9.59068
SC	6	70.65333±12.87967

Table S11 Changes in GSH concentration in serum of mice in each group (±SD)

Group	Sample size (cases)	GSH (ng/mL)
CON	6	1.12933±0.13179
DSS	5	0.86017±0.08698
PS	5	0.8965±0.08354
SS	6	0.93317±0.1267
SC	6	0.99233±0.13044

Table S12 Changes in MPO concentration in serum of mice in each group (±SD)

Group	Sample size (cases)	MPO (pg/mL)
CON	6	59.46183±17.73665
DSS	5	137.00367±21.9901
PS	5	121.67317±19.94566
SS	6	107.56317±26.67528
SC	6	87.49717±32.97999

Table S13 Changes in MDA content in serum of mice in each group (±SD)

Group	Sample size (cases)	MDA（nmol/mL）
CON	6	6.42067±1.0021
DSS	5	9.83617±0.92166
PS	5	8.21867±1.40761
SS	6	8.00533±1.35758
SC	6	7.89633±1.39656

Table S14 Permutational multivariate analysis of variance

method name	PERMANOVA
test statistic name	pseudo-F
sample size	30
number of groups	5
test statistic	2.79935
p-value	0.001
number of permutations	999
