# Supplementary figures and images for: Sea Buckthorn Fermented Milk with Lactiplantibacillus plantarum YHG-87 Mitigates Symptoms of DSS-Induced Ulcerative Colitis Disease in Mice
Source: Foods. 2025 Nov 5;14(21):3791. doi: 10.3390/foods14213791 (PMC12610435; doi:10.3390/foods14213791)

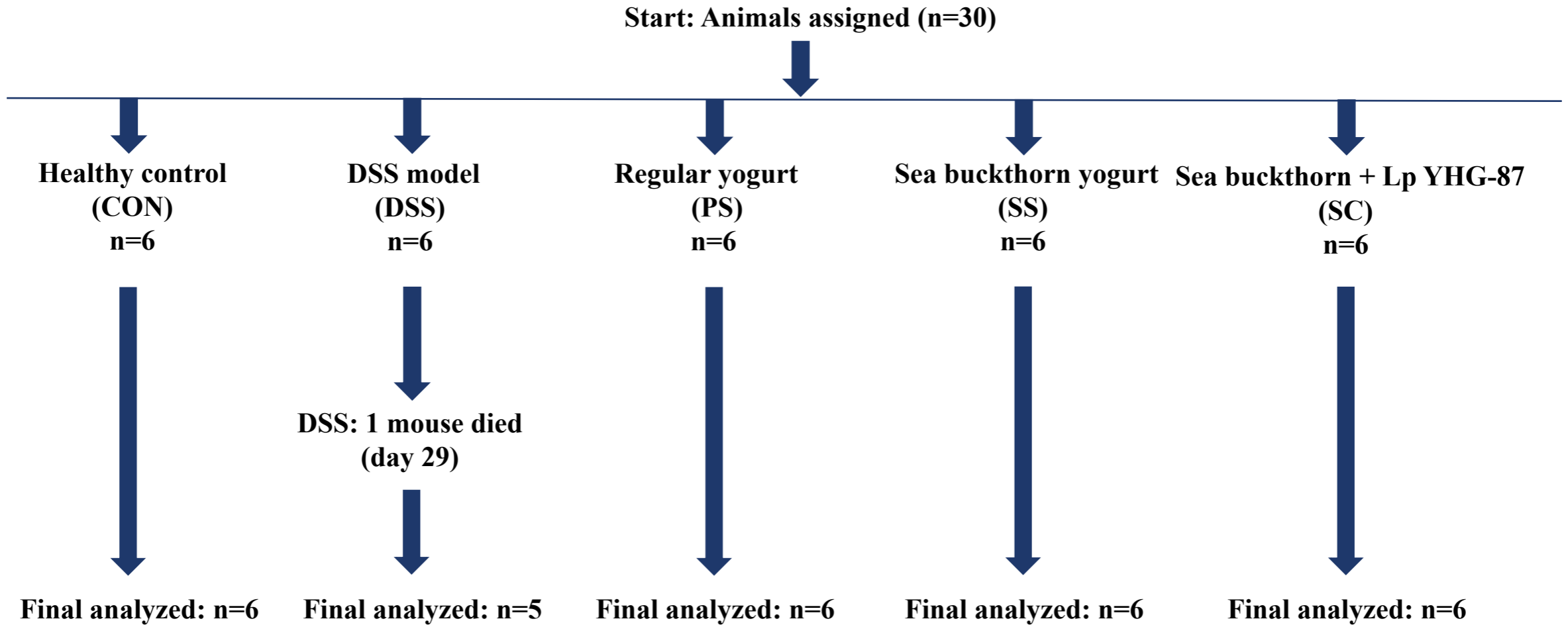

Supplement: Supplementary file 1 [file foods-14-03791-s001.zip › Figure S1.pdf]
